# Effect of Natural-Feeding Education on Successful Exclusive Breast-Feeding and Breast-Feeding Self-Efficacy of Low-Birth-Weight Infants

**Published:** 2014-01-05

**Authors:** Sibel Küçükoğlu, Ayda Çelebioğlu

**Affiliations:** Ataturk University, School of Nursing, Erzurum, Turkey

**Keywords:** Low Birth Weight, Infant, Neonate, Breast Feeding, Self Efficacy, Growth, Nursing

## Abstract

***Objective:*** The ideal nourishment for newborns with low birth-weight is breast milk. The purpose of the study was to determine the effect of natural-feeding education given to mothers of low-birth-weight infants, on the mothers’ breast-feeding self-efficacy level, breast-feeding success, and the growth of the infants.

***Methods:*** The study was conducted in a quasi-experimental way. The study group consisted of a total of 85 low-birth-weight infants and their mothers who had been treated in the neonatal clinics of two hospitals. The mothers included in the test group were given breast-feeding education for half an hour per day, during the first 5 days of their hospitalization. Home visits were carried out at the homes of the participants from both the test and control groups, until the infants reached 6 months of age. For collecting data, the following forms were used: Personal Information Form, Breast-feeding Self-Efficacy Form, LATCH Breast-feeding Assessment Tool, and Anthropometric Measurements Form. For analyzing the collected data, percentage, arithmetic mean, standard deviation, and chi-square we used, and, in independent groups, t-tests were employed.

***Findings*** ***:*** It was determined that natural-feeding education given to the mothers increases their breast-feeding self-efficacy levels and success in breast-feeding (*P*<0.05). It was found that in the test group, the rate of feeding the infants exclusively with breast milk is higher in comparison with the control group (*P*<0.001).

***Conclusion:*** Results of the study indicate that natural-feeding education increases breast-feeding self-efficacy levels, breast-feeding success, and the breast-feeding duration.

## Introduction

Breast-feeding with the proper technique, frequency, duration, exclusive breast-feeding up to 6 months of age, and continued breast-feeding along with appropriate complementary foods up to 2 years of age are the principal conditions of ideal nutrition for infants^[^^1^^]^. According to the data of the Turkish Population and Health Surveys, 68.9% of the newborns from 0 to 1 month of age, 42% of the infants between 4 and 5 months of age, and 21.9% of the infants between 4 and 5 months of age are exclusively fed with breast milk^[^^2^^]^. In Turkey, every year, approximately 1.4 million infants are born. According to the research, 10% of those infants are born with low-birth-weight^[^^3^^]^. 

 The ideal nourishment for newborns with low birth-weights is breast milk. The breast milk of mothers who gave birth to low-birth-weight infants is specially adjusted to the infants’ needs. Although low-birth-weight infants have sucking and swallowing difficulties, those who are born in the 32nd week of pregnancy or later can easily suckle breast milk^[^^4^^]^. However, nursing difficulties are still commonly seen in feeding such infants for reasons related to mothers, such as their doubting that their milk will suffice, lacking the support of healthcare personnel, and having incorrect traditional beliefs^[^^5^^]^. Although the fact that low-birth-weight infants have a greater need for breast milk in comparison to infants with normal birth-weight, they are more frequently and more commonly bottle-fed with artificial formulas^[^^1^^]^. The studies carried out in this field exhibited that the breast-feeding rates of mothers of low-birth-rate infants are significantly lower than that of those who had gave birth at term^[^^6^^]^. In addition it was found that the breast-feeding duration of the low-birth-weight and preterm infants is considerably shorter than that for full-term normal-weight infants^[^^7^^,^^8^^]^. 

 Perception of self-efficacy plays an important role in determining the actions an individual will take or prevent. Breast-feeding self-efficacy is the confidence the mother feels pertaining to breast-feeding^[^^9^^]^. It was determined that while mothers with low breast-feeding self-efficacy tend to wean far earlier than the recommended period, mothers having high breast-feeding self-efficacy experience fewer problems in starting and sustaining breast-feeding^[^^10^^]^. 

 It was determined that the actions of healthcare professionals working in newborns’ units of hospitals, such as enlightening mothers of low-birth-weight infants on breast-feeding, encouraging them to participate in their babies’ care, giving them emotional support, and conducting regular follow-up home visits after their release from hospital, increase the mothers’ postpartum self-efficacy and breast-feeding success^[^^11^^,^^12^^]^. No study that examines the effects of natural-feeding states of the mothers of low-birth-weight infants, their self-efficacy levels, and the interferences to promote natural feeding on breast-feeding success could be found in Turkey. 

 The present study was conducted with the purpose of determining the effect of natural-feeding education given to the mothers of low-birth-weight infants treated in newborns’ clinics, on the mothers’ breast-feeding self-efficacy levels, and the breast-feeding success of the infants.

## Subjects and Methods

The following is a description of the subjects and methods of the study.

**Universe and Sample of the Study**

The universe of the study, which was carried out between January 2010 and January 2011, was comprised of the low-birth-weight infants and their mothers, who received treatment in the neonatal clinics of two hospitals located in the eastern Turkey. Families included in this study had lower levels of income and education, according to the average levels of income and education in Turkey. The region of the study has a cold climate and hard geographical conditions compared to other regions. However, the region where the study was carried out is an important health center that accepts patients from other provinces. The sample size of the study was determined through power analysis. The sample of the study consisted of 86 low-birth-weight infants and their mothers, with 42 for test group and 44 for control group, who were selected from the mentioned universe by means of the nonprobability accidental sampling method. The sample size for the study, 95% test power with the effect size of 0.8 represents the power of the universe, and 85% were identified as a result of the power analysis. In order to prevent the mothers from influencing each other with the education they received, first, the data of the control group, and later, the data of the test group were collected. 

**Inclusion Criteria**

Two sets of criteria were considered in the study: 

infant-related criteria and mother-related criteria.

Infant-related criteria: Infants who had following criteria were included in the study:

- had no conditions preventing nutrition,
- had birth weight of less than 2500 g,
- were born at more than 32 weeks of gestational age, and
- were hospitalized for at least 5 days

Mother-related criteria: Mothers who had following criteria were included in the sample:

- were between the ages of 18 and 35,
- had no seeing or hearing problems,
- were living in the city center, and
- were open to communication and cooperation

Examining the demographic attributes of the mothers and infants included within the scope of the study (mother's age, level of education, employment status, income level, whether or not the pregnancy was planned, number of children, delivery method, breast-feeding experience, breast-feeding education, duration she intends to breastfeed her baby, gender of the baby, gestational week of birth, and when breast-feeding started) showed that there is no significant difference between the groups (*P*>0.05) and that both groups were similar in terms of demographic attributes (Table 1).

**Data Collection Tools**

For collecting the data the “Personal Information Form,” “Breast-feeding Self-Efficacy Form,” “LATCH Breast-feeding Assessment Tool,” “Breast-feeding Follow-Up Card,” “Infant Anthropometric Measurement Form,” infant weighing scale, infant height measurement ruler, and head circumference tape were used. 

*Personal Information Form: *The Personal Information Form used in data collection consists of 13 questions focused on the information pertaining to the mother (mother’s age and employment status and the income level of the family), information about the pregnancy (planning of pregnancy, number of children, and delivery method) information on the newborn (gender and gestational week) and breast-feeding-related information (breast-feeding experience, if breast-feeding education was received, when breast-feeding started, and intention regarding feeding exclusively with breast milk).

*Breast-feeding Self-Efficacy Scale: *The initial form of the Breast-feeding Self-Efficacy Scale, which was developed by Dennis^[^^13^^]^ (2003) for the purpose of assessing mothers’ breast-feeding self-efficacy, had 33 items.

**Table 1:** Comparison of experimental and control group characteristics of mothers regarding their breastfeeding and demographic features

**Variable**	**Features**		**Experimental ** **Group [** **n (%)]**	**Control Group**	**Total**	***P.*** ** value**
				**n (%)**	**n (%)**	
**Age group (year)**	19-29		30 (71.4)	28 (65.1)	58 (68.2)	0.5
	30 and above		12 (28.6)	15 (34.9)	27 (31.8)	
**Education**	Primary education		21 (50.0)	23 (53.5)	44 (51.8)	0.7
	High school		14 (33.3)	11 (25.6)	25 (29.4)	
	University		7 (16.7)	9 (20.9)	16 (18.8)	
**Work status**	Housewife		36 (85.7)	36 (83.7)	72 (84.7)	0.8
	Working		6 (14.3)	7 (16.3)	13 (15.3)	
**Income status**	Less expense to income		1 (2.4)	5 (11.6)	6 (7.1)	0.2
	Expense to be equivalent income		23 (54.8)	21 (48.8)	44 (51.8)	
	More income to expense		18 (42.9)	17 (39.5)	35 (41.2)	
**Pregnancy planning status**	Planned		36 (85.7)	35 (81.4)	71 (83.5)	0.6
	Not planned		6 (14.3)	8 (18.6)	14 (16.5)	
**Number of children**	First		18 (42.9)	17 (39.5)	35 (41.2)	0.7
	Two or more		24 (57.1)	26 (60.5)	50 (58.8)	
**Mode of birth**	Normal		17 (40.5)	16 (37.2)	33 (38.8)	0.8
	Cesarean		25 (59.5)	27 (62.8)	52 (61.2)	
**Mother Breastfeeding ** **Experience**		Yes	24 (57.1)	26 (48.8)	50 (58.8)	0.5
		No	18 (42.9)	17 (51.2)	35 (41.2)	
**The condition of ** **breastfeeding Education**		Yes	8 (19.0)	7 (16.3)	15 (17.6)	0.7
		No	34 (81.0)	36 (83.7)	70 (82.4)	
**Time for giving breast milk**	4 months		17 (40.5)	19 (44.2)	36 (42.4)	0.7
	6 months		15 (35.7)	12 (27.9)	27 (31.8)	
	Mixed (breast milk+ mama)		10 (23.8)	12 (27.9)	22 (25.9)	
**Sex of the baby**	Female		28 (66.7)	26 (60.5)	54 (63.5)	0.5
	Male		14 (33.3)	17 (39.5)	31 (36.5)	
**Gestational week**	32-35		13 (31.0)	15 (34.9)	28 (32.9)	0.8
	36-38		24 (57.1)	22 (51.2)	46 (54.1)	
	39 and above		5 (11.9)	6 (14.0)	11 (12.9)	
**Baby’ first breastfeeding time**	Shortly after birth		8 (19.0)	4 (9.3)	12 (14.1)	0.5
	Within 60 minutes		13 (31.0)	11 (25.6)	24 (28.2)	
	61 minutes or more		6 (14.3)	7 (16.3)	13 (15.3)	
	Breastfeeding did not happen		15 (35.7)	21 (48.8)	36 (42.4)	

Later, in 2003, a 14-item short form of the scale was developed. Dennis suggests this short form for use. While the lowest point that can be scored in the scale is 14, the maximum is 70. Higher points scored indicate high breast-feeding self-efficacy. 

 The validity and reliability study of the scale for the Turkish was carried out by Tokat^[^^10^^]^ (2009), and its Cronbach’s alpha value was found to be 0.86. In the present study, the Cronbach’s alpha value of the scale found for immediate postpartum was 0.74, and it was 0.98 by the end of the 6th month following the birth.

*LATCH Breast-feeding Assessment Tool: *The LATCH Breast-feeding Assessment Tool was developed by Jensen et al^[^^14^^]^. Turkish validity of the assessment tool was examined two times, and it was found to be a reliable tool^[^^17^^,^^18^^]^. The Cronbach’s alpha value of the LATCH Breast-feeding Assessment Tool was found to be 0.95, by Yenal and Okumus^[^^15^^]^, and 0.96 by Koyun^[^^16^^]^. In the present study, the Cronbach’s alpha value of the LATCH Breast-feeding Assessment Tool was found to be 0.93. 

 Whereas the maximum point that can be scored in the LATCH Breast-feeding Assessment Tool is 10, the minimum is 0^[^^15^^,^^16^^]^. Higher points scored in the tool indicate higher success in breast-feeding.

*Breast-feeding Follow-Up Form*: This is a form developed by the researcher to assess breast-feeding activities observed through the LATCH breast-feeding scoring system and to record the results.

**Data Collection**

The researcher collected the data by conducting one-on-one interviews with the mothers in hospitals and in their homes.

*Collection of Pretest Data: *In order to collect pretest data, on the date of their hospitalization, the mothers of low-birth-weight infants of both test and control groups were given the Personal Information Form, Breast-feeding Self-Efficacy Scale and LATCH Breast-feeding Assessment Tool.

*Collection of Posttest Data: *For collecting posttest data, the mothers of both test and control groups were visited once in the hospital on the 5th day of their hospitalization and then once a month in their homes for a period of 6 months. During the visits, the mothers were asked to breastfeed their babies. By observing their breast-feeding statuses, the Breast-feeding Follow-Up Forms were filled. At the end of the 6th month, the Breast-feeding Self-Efficacy Scale and the LATCH Breast-feeding Assessment Tool were once again applied at the mothers’ home. During the home visits, necessary reminders were made to the mothers of the test group, and their questions were answered. The visits made to the homes of the participants of the test group were limited to 30 minutes, whereas they were limited to 10 minutes for the control group. 

 Immediately after the application of the final tests at the end of the 6th month, the mothers in the control group were provided with an informative booklet.

**Interference Tools**

The following is a description of the interference tools used during the study.

Educatory booklet – *Every Mother Can Breastfeed; Wanting to Is Enough*

The educatory booklet covers information regarding the importance of self-efficacy in breast-feeding and of breast milk for infants with low birth-weight, the things breast-feeding and breast milk are good for, preparation of the mother for breast-feeding, risks of artificial feeding, breast-feeding positions, breast-feeding duration and intervals, nutrition of the nursing mothers, extraction and preservation of breast milk, feeding methods of low-birth-weight infants, and the most common problems the mothers experience in the process of starting and sustaining breast-feeding. In addition, the participant mothers received no formula-feeding training or information, and no instruction was given to them at the hospitals because these hospitals were “baby-friendly” hospitals. 

**Low-Birth-Weight Infant Dummy**

A training dummy of approximately 750 g weight, with movable head, neck and limbs for representing a low-birth-weight infant was used in the study for training purposes. 

**Breast-feeding Pillow**

The pillow used in the study was designed for comfortable use during breast-feeding. The pillow was 150 cm long, 25 cm wide, and 8 l in volume. Produced with materials that are free of carcinogenic and allergenic substances, the pillow was made of 100% cotton, with a soft, light, washable cover. In order to prevent infections, the cover of the pillow was changed with a new one before each breast-feeding session.

**Nursing Interference**

For 5 days starting from their first day of hospitalization, the mothers included in the test group were given education in line with the content of the book titled *Every Mother Can Breastfeed; Wanting to Is Enough*, at the hours convenient for the mothers and their babies. At the end of each training session, breast-feeding was first demonstrated by the researcher with the use of the breast-feeding pillow and the training dummy. Afterwards, the mothers were first asked to practice breast-feeding with the dummy, and then asked to breastfeed their own babies. At the end of 5 days of training, which took about 30 minutes every day, the mothers in the test group were provided with the booklet *Every Mother Can Breastfeed; Wanting to Is Enough*. One week after their release from the hospital, the mothers of the test group were contacted via phone and asked whether they were having any problems regarding breast-feeding. They were informed again regarding their inadequate or wrong practices.

 During the study period, the nurse who worked day-shift provided breast-feeding counseling in line with the hospital policy, but the counseling could not be given daily and regularly, and no equal amount of counseling-time was given for each patient. The counseling did not include home visits after hospital discharge at all. Yet, our counseling was regularly provided 5 days a week, with demonstrations on baby-dolls, and mothers were made to practice. In addition, monthly regular home visits were paid to the babies. As for the control group, they were given only breast-feeding training by the breast-feeding trainer. 

**Evaluation of Data**

Evaluation of the data obtained from the study was carried out in a computerized environment with the use of the SPSS 15.0 (Statistical Package for Social Science) package software. In the evaluation of the data, percentage distribution, chi-square test, t-test, and Cronbach’s alpha coefficient calculations were used. The significance value has been established as *P*<0.05 in this study.

**Ethical Principles of the Study**

Before commencing the study, both written and verbal permissions of the hospitals, in which the study was to be carried out, were obtained. The study was also submitted to the ethical committee of the university, where the study was to be conducted, and the approval was granted. Before collecting any data, the purpose and duration of the study, and the things expected to be realized within the scope of the study were clearly explained to the mothers; their questions were answered, and then their written and verbal consents were obtained.

 The hospitals that were not “baby friendly” were excluded from the study and were asked to provide routine breast-feeding counseling for the control group, too.

## Findings

As for the intergroup–intragroup comparison of the breast-feeding self-efficacy point averages of the test and control groups, it was found that the posttest point average of the mothers included in the test group was considerably higher than the posttest point average of the mothers from the control group, and that difference was statistically significant (*P*<0.001, Table 2).

**Table 2:** Intra-group and intergroup comparison of breast feeding self-efficacy scale

**Breast Feeding Self-Efficacy Scale **	**Pretest**	**Posttest**	**t**	***P. *** **Value**
	**Mean (SD)**	**Mean (SD)**		
**Test group**	42.00 (9.99)	65.81 (7.37)	12.617	<0.001
**Control Group**	41.77 (11.40)	39.70 (12.21)	0.898	0.4
**t**	0.100	11.898		
***P. *** **Value**	0.9	<0.001		

SD: Standard Deviation

**Table 3:** Comparison of the LATCH breastfeeding assessment tool point averages scored of mothers

**Tagged with** **Day** **and** **Month**	**Test Grup** **Mean (SD)**	**Control Grup** **Mean (SD)**	**t**	***P. *** **value**
**First day of hospitalization**	5.90 (1.62)	5.81 (1.72)	0.060	0.8
**5** ^th^ ** day**	8.76 (1.90)	6.35 (2.29)	5.287	<0.001
**1** ^st^ ** month**	9.60 (1.23)	7.65 (2.05)	5.293	<0.001
**2** ^nd^ ** month**	9.79 (0.95)	8.19 (1.94)	4.803	<0.001
**3** ^rd^ ** month**	9.83 (0.93)	8.12 (2.24)	4.595	<0.001
**4** ^th^ ** month **	9.86 (0.93)	7.67 (.41)	5.492	<0.001
**5** ^th^ ** month**	9.86 (0.93)	7.53 (2.40)	5.851	<0.001
**6** ^th^ ** month**	9.86 (0.93)	7.51 (2.42)	5.567	<0.001

SD: Standard Deviation

In the intergroup comparison of the point averages of mothers from both the test and control groups obtained from the LATCH Breast-feeding Assessment Tool, it was determined that the LATCH point averages of the test group mothers increased from the control group scored (*P*<0.005, Table 3). While there were no babies that refused breast milk or “weaned prematurely” from the test group, it was determined that 85.7% of the babies were being fed exclusively with breast milk and that there was a statistically significant difference between the test and control groups in these terms (*P*<0.001, Table 4).

## Discussion

Despite the facts that the importance of breast milk in infant nutrition is emphasized at both national and international levels, and that it is accepted by many countries and announced in several declarations that healthy nutrition is a right for all children, it is believed that throughout the world today only 39% of the newborns are exclusively fed with breast milk in the first 6 months of life^[^^17^^]^. According to the data of the TNSA ^[^^2^^]^, in Turkey, 68.9% of the newborns from 0 to 1 month of age, 42% of the infants between 4 and 5 months of age, and 21.9% of the infants between 4 and 5 months of age are exclusively fed with breast milk. 

 At the end of the study, it was understood that the mothers from the test group had considerably higher perceptions of self-efficacy, and they experienced fewer breast-feeding problems in comparison to those in the control group (Table 2). There are many studies emphasizing that the education and training offered regarding breast-feeding have positive effects on mothers’ willingness to breastfeed^[^^2^^,^^10^^,^^18^^,^^19^^]^, and, that as a modifiable perception, breast-feeding self-efficacy can be increased^[^^11^^,^^13^^,^^20^^]^. In the literature it is stated that the high breast-feeding self-efficacy level of the mother has considerable effects on her behavior of sustaining breast-feeding^[^^12^^,^^16^^,^^20^^]^. However, Barnes and Adamson^[^^21^^]^ (2004) determined in their study that mothers of preterm infants have considerably low perceptions of self-efficacy, and these mothers need much more support.

**Table 4:** Distribution of the ways with which the babies from test and control groups were being fed at the end of the sixth month

**Nutrition Type**	**Test Group**	**Control Group**	**t ** ***P. *** **value**
	**n (%)**	**n (%)**	
**Only breast milk**	36 (85.7)	5 (11.6)	<0.001
**Mixed feeding**	6 (14.3)	22 (51.2)	<0.001
**Milk** **was interrupted** **before** **the sixth month**	0	6 (14.3)	<0.001
**Did not take breast milk**	0	7 (16.3)	<0.001

The breast-feeding successes of the mothers from both test and control groups in the 6 months of the study exhibited that the LATCH points of the test group are higher than those of the control group and that the difference between the groups is statistically significant (Table 3). The literature has many studies indicating that education has an important effect on breast-feeding^[^^10^^,^^12^^,^^22^^,^^23^^]^. 

 In the study it was found that 85.7% of the mothers of the test group sustained exclusive breast-feeding during the 6 months following their release from the hospital, whereas only 38.9% of the mothers from the routine control group sustained exclusive breast-feeding in the same period (Table 4). The reason why the mothers from the test group sustained exclusive breast-feeding for a longer period than the control group did, is believed to be because within the first 5 days following the birth, mothers from the test group were given education by the researcher on breast-feeding techniques and the benefits of breast milk, which were controlled and supported again after 1 week, and the education was maintained through the 6 months postpartum. 

 In previous research, it was determined that the breast-feeding periods of low-birth-weight and premature infants are considerably lower than those of normally born infants^[^^7^^,^^8^^]^. Moreover, in another study carried out in this field, it was exhibited that the breast-feeding durations of mothers of low-birth-weight infants are significantly lower than those who had given birth at term^[^^6^^]^. In their study carried out with the participation of mothers of low-birth-weight infants, Santoro-Junior and Martinez^[^^24^^]^ (2007) determined that the education and support given to the mothers has positive effects on the duration of feeding their babies exclusively with breast milk. In addition, the study of Varol and Yıldız^[^^12^^]^ (2006) emphasized that breast-feeding education and follow-up maintained for 6 months starting from the date of birth increases the duration of exclusive breast-feeding. Koskinen et al^[^^25^^]^ confirmed that early breast-feeding experiences and maternity hospital practices have an association with maternal breast-feeding self-efficacy. Masters et al^[^^26^^]^ determined that the program (a theory-based booklet and pre- and postnatal home visits by trained assistants) significantly improved exclusive breast-feeding rates at 6 months postpartum. 

 Because the hospital where the study was conducted was a baby-friendly hospital, no training and guidance was given to the mothers about formula feeding. That babies who received formula feeding or mixed feeding had lower weights in our study, may have resulted from the fact that mothers did not receive any training about formula feeding before hospital discharge and they tried to feed their babies according to their feeding-standards.

## Conclusion

Breast-feeding education and home visits are affective for the promotion and implementation of breast-feeding. Our results show that an interactive education can increase the mother’s knowledge, management of breast-feeding practice, breast-feeding self-efficacy, and baby growth. The effect of introducing an interactive supportive breast-feeding education program may well be stronger in countries with less developed health care systems. It is necessary to increase the awareness of the nurses by providing them with on-the-job trainings so that they can train mothers and fathers who have low-birth-weight infants, about the importance of breast-feeding self-sufficiency and breast milk. Moreover, having nurses who are exclusively breast-feeding trainers will increase the quality of the training for mothers.
